# Characterization of Three L-Asparaginases from Maritime Pine (*Pinus pinaster* Ait.)

**DOI:** 10.3389/fpls.2017.01075

**Published:** 2017-06-23

**Authors:** Sonia H. Van Kerckhoven, Fernando N. de la Torre, Rafael A. Cañas, Concepción Avila, Francisco R. Cantón, Francisco M. Cánovas

**Affiliations:** Departamento de Biología Molecular y Bioquímica, Facultad de Ciencias, Universidad de MálagaMálaga, Spain

**Keywords:** conifers, maritime pine, asparagine, aspartate, nitrogen metabolism, proteolytic processing

## Abstract

Asparaginases (ASPG, EC 3.5.1.1) catalyze the hydrolysis of the amide group of L-asparagine producing L-aspartate and ammonium. Three ASPG, PpASPG1, PpASPG2, and PpASPG3, have been identified in the transcriptome of maritime pine (*Pinus pinaster* Ait.) that were transiently expressed in *Nicotiana benthamiana* by agroinfection. The three recombinant proteins were processed *in planta* to active enzymes and it was found that all mature forms exhibited double activity asparaginase/isoaspartyl dipeptidase but only PpASPG1 was able to catalyze efficiently L-asparagine hydrolysis. PpASPG1 contains a variable region of 77 amino acids that is critical for proteolytic processing of the precursor and is retained in the mature enzyme. Furthermore, the functional analysis of deletion mutants demonstrated that this protein fragment is required for specific recognition of the substrate and favors enzyme stability. Potassium has a limited effect on the activation of maritime pine ASPG what is consistent with the lack of a critical residue essential for interaction of cation. Taken together, the results presented here highlight the specific features of ASPG from conifers when compared to the enzymes from angiosperms.

## Introduction

Asparaginases (ASPG, EC 3.5.1.1) catalyze the hydrolysis of the amide group of L-asparagine (Asn) producing L-aspartate (Asp) and ammonium. Some ASPG are also able to hydrolyze modified substrates such as glycosylated L-Asn or β-aspartyl dipeptides, resulting in the release of L-Asp. In 1980, Sodek reported the presence of two different ASPG activities in plants, dependent or independent of potassium ion (K^+^) for their activity. Bruneau et al. (2006) confirmed the co-existence of two ASPG in *Arabidopsis thaliana* and it was initially observed that the K^+^-dependent type is strictly specific for Asn whereas the K^+^-independent ASPG can also accept isoaspartyl dipeptides as substrates. Similar results were found in *Lotus japonicus* (Credali et al., 2011), but recent work on the *Arabidopsis* ASPG also reports a double activity asparaginase/isoaspartyl dipeptidase for the K^+^-dependent type (Gabriel et al., 2012). Crystallographic structure analysis of a K^+^-dependent ASPG from *Phaseolus vulgaris* showed a catalytic pocket big enough to host substrates larger than Asn, but it has been proposed that substrate specificity is probably determined by the nature of the amino acids in the vicinity of the substrate side chain(Bejger et al., 2014). The existence of these two subgroups within plants suggests a functional specialization for the isoforms.

Plant ASPG belong to the superfamily of Ntn (N-terminal nucleophile) hydrolases which include enzymes with large diversity in catalytic activity (Galperin and Koonin, 2012) and different oligomeric forms (Michalska and Jaskólski, 2006). Members of this protein family share a typical fold which incorporates a nucleophilic residue (serine, threonine, or cysteine) at the N-terminal incorporated in a β-sheet, providing the capacity for a nucleophilic attack and the possibility of autocatalytic processing (Brannigan et al., 1995). Plant ASPG, as well as aspartylglucosaminidase and taspase1, occur in a heterotetrameric (αβ)_2_ structure which results from the combination of two autocatalytically cleaved precursors into their α and β subunits (Michalska and Jaskólski, 2006).

The autocatalytic activation reaction is a posttranslational modification *in cis* which, apart from the nucleophile, requires a precise alignment of several functional groups (Guan et al., 1996; Xu et al., 1999). When comparing protein sequences of ASPG isoforms and homologs in plants, high diversity is noticed in the region preceding the catalytic nucleophile, both in sequence as well as in length. This region, often referred to as a variable loop (VL), presents a lack of electron density in X-ray diffraction data of the protein crystals and therefore probably does not have a defined secondary structure (Michalska et al., 2006; Bejger et al., 2014). This has made it difficult to postulate a hypothesis about its function based on structural aspects. However, there are evidences that this region may have a role in autocatalytic processing and substrate specificity. In particular, reciprocal exchange of the C-terminal end of the α subunit between *Arabidopsis* K^+^-dependent and K^+^-independent enzymes affected the affinity for Asn and β-Asp-His, and deletions in this region impacted self-cleavage processing and catalytic activity (Gabriel et al., 2012). It has been postulated that the autocatalytic activation mechanism and amino acid residues involved differ between the ASPG of different species and possibly a certain flexibility in the region that precedes the nucleophile is necessary (Michalska et al., 2008; Nomme et al., 2012). Recombinant plant ASPG expressed in *E. coli* have been found to mature more slowly but the autocatalytic activation process continues *in vitro* (Michalska et al., 2006; Cantor et al., 2009; Nomme et al., 2012). *Pinus sylvestris* ASPG, however, did not present autoproteolytic capacity *in vitro* but the addition of root extracts from young trees resulted in enzyme activation (Cañas et al., 2007). These findings suggest a different maturation mechanism for these enzymes in gymnosperms, which could require the assistance of an external factor. The primary structure of the *P. sylvestris* ASPG, when compared to that of angiosperms, is about 50 amino acids larger due to a non-conserved sequence located in the VL.

Asn is a metabolite used by plants as a vehicle for storage and transport of nitrogen (N) (Borek and Jaskólski, 2001) and, therefore, as a N donor in sink tissues through ASPG activity (Sieciechowicz et al., 1988; Gaufichon et al., 2013). The use of this N donor differs temporally and between species, organs, and tissues (Lea et al., 2007). On the other hand, Asp is an immediate precursor for the biosynthesis of other amino acids of the Asp family (lysine, threonine, methionine) (Azevedo et al., 2006) which can also be substrates for further metabolic reactions that produce isoleucine, glycine, and serine (Hildebrandt et al., 2015). ASPG activity therefore could contribute in providing substrates for several metabolic pathways involved in development and growth. In the hypocotyls of pine seedlings, Asn seems to be the main N storage molecule during early pine development and a temporal and spatial correlation between ASPG expression and appearance of the vascular system in pine suggests a function for this enzyme in N mobilization to sustain the development of this structure (Cañas et al., 2007). Thus, asparaginase appears to play an important role during development of the xylem in the hypocotyl once the pine seedlings have consumed the seed reserves. This is essential for distribution of water and nutrients from the roots and therefore for seedling survival after germination.

*Pinus pinaster* Aiton is one of the most common conifer species in the occidental Mediterranean basin where it is of great ecological importance, but also highly valued for the production of wood and resin. Pine trees are tolerant to diverse types of stress and are able to grow on poor soil and problematic habitats (Lev-Yadun and Sederoff, 2000). Compared to other pine species, *P. pinaster* populations present a high rate of genetic variability, polymorphisms and heterozygosity (Salvador et al., 2000) which contributes to its value as a model species and facilitates identification of suitable genotypes for domestication. In this work, we present the biochemical characterization of pine asparaginases and new insights into the function of the VL sequence in conifers.

## Materials and Methods

### Biological Material

*Escherichia coli* strains DH5α and BL21-AI (Invitrogen Corporation, Carlsbad, CA, United States) were used for plasmid propagation and expression of the protein in a prokaryotic system, respectively. For protein production in *Nicotiana benthamiana*, *Agrobacterium tumefaciens* strain C58C1 was applied. *P. pinaster* seeds from Sierra Bermeja provenance (Estepona, Spain) were germinated and grown as previously described (Cañas et al., 2007).

### RNA Extraction and cDNA Synthesis

Tissue samples were ground under liquid nitrogen with a mortar and pestle. Total RNA was extracted from 100 mg ground powder as previously described (Canales et al., 2012). The integrity of RNA in the samples was verified by agarose gel electrophoresis.

To obtain the full-length cDNA for the *PpASPG1* gene, cDNA was generated following the indications of the “FirstChoice RLM RACE” kit (Applied Biosystems/Ambion, Austin, TX, United States) using 10 μg of total RNA extracted from xylem as a template. For amplification of the *PpASPG2* and *PpASPG3* cDNAs 1 μg of total RNA extracted from radicles of seedlings was used to synthesize single strand cDNA using iScript cDNA Synthesis kit (Bio-Rad, Hercules, CA, United States).

### PCR Amplification and Cloning of *ASPG* cDNAs

All PCR reactions were carried out with iProof High-Fidelity DNA Polymerase (Bio-Rad, Hercules, CA, United States). Reverse primers A1-1 and A1-2 (see Supplementary Table S1) were designed on a previously known EST sequence (Villalobos et al., 2012) to amplify the 5′UTR region of the *PpASPG1* gene using 5′RACE cDNA template. PCR reactions were performed under the following conditions: initial denaturalization for 2 min at 95°C, 30 cycles of 30 s at 95°C, 30 s at 60°C and 1 min 15 s at 72°C with a final extension of 10 min at 72°C. The amplified DNA fragment was blunt end ligated in the vector pBluescript SK+ (Agilent Technologies, Santa Clara, CA, United States) and propagated by transforming *E. coli.* Inserts were verified by sequencing with a Beckman Coulter CEQ8000 sequencer. Forward primer A1-3 was designed over the 5′UTR region to obtain the full-length cDNA using the 3′RACE cDNA as a template. The PCR product was also ligated into vector pBSK and transformed into *E. coli* strain DH5α competent cells for propagation and sequencing. The final plasmid preparation was used as a template for constructions in expression vectors.

Primers A1-4 and A1-5 were used to amplify the complete ORF of *PpASPG1* without any tags, incorporating a unique restriction site for *PacI* in the N-terminal region and a *NotI* site in the C-terminal region. cDNAs were amplified using primers A2-1 and A2-2 for *PpASPG2* and primers A3-1 and A3-2 for *PpASPG3* and cDNA of young pine plantlets as a template. Primers were designed on the UTR regions of unigenes 15077 and 36994 available at the Sustainpine V.3.0 database (Canales et al., 2014). A second PCR reaction was carried out to amplify the ORF and to incorporate the restriction sites for *PacI* and *NotI* to facilitate the cloning in the expression vector pJL-TRBO, as well as an HA and poly-His tag at the C-terminal, using primers A2-3 and A2-4 for *PpASPG2* and primers A3-3 and A3-4 for *PpASPG3.*

### Generation of Deletion Constructs by PCR

To generate *PpASPG1* cDNAs with different deletions in the internal variable region, sets of two deletion primers with complementary sequences were designed. Half of the sequence of each primer was derived from 5′-flanking region of the sequence to be eliminated and the other half from the 3′-flanking region. Deletion primers A1-6 and A1-7 were used for D1, A1-8 and A1-9 for D2 and A1-10 and A1-11 for D3 construction (see Supplementary Table S1 for primer sequences). For every deletion two rounds of PCR were carried out. In the first round, two PCR reactions were performed, each one with one primer of the corresponding pair of complementary deletion primers and a second primer derived from one end of the complete ORF sequence (A1-4 forward and A1-12 reverse primers). This procedure was used to separately amplify the fragments covering the sequence for the α and β subunit (for example, for deletion D1, A1-6 was combined with A1-12 to produce the β-subunit encoding fragment and A1-7 with A1-4 to produce the α-subunit encoding fragment). Consequently, the 3′-end of the amplified sequence for the α subunit and the 5′-end of the sequence for the β subunit had the same sequence, corresponding with the sequence of the primers A1-6 and A1-7 for D1, A1-8 and A1-9 for D2 and A1-10 and A1-11 for D3. For each deletion, the products of the first PCR reactions were then combined as template in a second round of PCR, and amplified with the primers A1-4 and A1-12. After denaturing and annealing in the first PCR cycle, sense DNA strands derived from the sequence for the α subunit can be annealed with the antisense DNA strand derived from the sequence for the β subunit through the complementary region introduced by the deletion primers, and each strand will act as a primer for the synthesis of the complementary strand by the DNA polymerase. This new product consisting in the precursor coding sequence, but with the selected internal sequence deleted, was amplified in the subsequent PCR cycles with the A1-4 and the A-12 primers. The A1-4 forward primer included a *Pac*I restriction site and the A-12 reverse primer a *Not*I restriction site to facilitate the cloning in the expression vector pJL-TRBO. The reverse primer A1-12 also includes a C-terminal HA and poly-His tag sequence. For comparison, the complete sequence of the *PpASPG1* ORF was also amplified with these primers to include the tag.

### Construction of Recombinant Expression Vectors for *In planta* Expression

For *in planta* expression, the different PCR products derived from the ORFs described above were digested with restriction enzymes *Pac*I and *Not*I Fast Digest (Thermo Fisher Scientific Inc., Waltham, MA, United States) and ligated into the pJL-TRBO vector (Lindbo, 2007) previously digested with the same restriction enzymes.

### *In planta* Protein Expression, Extraction, and Western Blot Analysis

*Agrobacterium tumefaciens* strain C58C1 was transformed with the expression vector pJL-TRBO by electroporation. Overnight grown cells harvested by centrifugation were used to infiltrate *N. benthamiana* leaves with a syringe by applying slight pressure on a small incision on the abaxial side of the leaves. Five days later the infiltrated areas of the *N. benthamiana* leaves were homogenized in liquid nitrogen. Extraction buffer [50 mM Hepes pH 7.8, 50 mM KCl, 5 mM EDTA, 10% glycerol (v/v), 10 mM 2-mercaptoethanol and 1 mM PMSF] was added in proportion 2:1 (v/w). Crude extracts were obtained by centrifugation at 18,660 ×*g* at 4°C for 20 min. Successful expression *in planta* was confirmed by western blot analysis and asparaginase activity of crude extracts. A negative control consisting in crude extracts from *N. benthamiana* leaves infiltrated with *A. tumefaciens* transformed with the empty pJL-TRBO vector was always included as a reference.

Laemmli buffer (Laemmli, 1970) as well as 0.5 mM 2-mercaptoethanol were added to the crude extracts and proteins resolved on 12.5% (w/v) polyacrylamide gels. The gels were transferred onto nitrocellulose membranes (Whatman GmbH, Dassel, Germany) and the presence of ASPG polypeptides was immunorevealed as described previously (Cañas et al., 2007), using either the antiserum raised against pine ASPG or an antibody against the HA tag. Detection of immunocomplexes was carried out by chemiluminescence using the SuperSignal West Pico Chemiluminescent Substrate system (Thermo Fisher Scientific Inc., Waltham, MA EE.UU., United States). Protein levels were determined by the Bradford procedure (Bradford, 1976).

### Protein Purification of Recombinant Proteins Produced *In planta*

The PpASPG1 and PpASPG1-D3 recombinant proteins were purified from *N. benthamiana* leaf extracts mostly as described by Sodek and Lea (1993) with the following exceptions and specifications. Soluble proteins extracted from the infiltrated tobacco leaf areas were concentrated by the gradual addition of (NH4)_2_SO_4_ up to 80% saturation. The pellet was resuspended in extraction buffer, ethanol was added up to a concentration of 60% (v/v), and incubated on ice for 1 h. After centrifugation at 2,000 ×*g* for 20 min at 4°C insoluble proteins were discarded and ASPG was recovered in the supernatant. The mixture was extensively dialyzed in extraction buffer to remove the ethanol, after which the protein was concentrated by reverse dialysis in sucrose. The concentrate was loaded onto a DEAE-Sephacel (GE Healthcare Bio-Sciences AB, Uppsala, Sweden) 50 mL column. Elution was performed with a gradient formed of 50 mM KCl and 500 mM KCl at a flow rate of 42 mL/h. The fractions with ASPG activity were pooled and concentrated by reverse dialysis in sucrose before subjection to size-exclusion gel chromatography on a Sephacryl S-300 column (Sigma-Aldrich Co. LLC. St. Louis, MO, United States) (93 cm × 2 cm) at a flow rate 16 mL/h. Fractions with ASPG activity were pooled, flash-frozen in liquid nitrogen and stored at -80°C until further use.

PpASPG2 and PpASPG3 recombinant proteins were purified from the leaf extracts applying Protino^®^ Ni-TED 2000 columns (MACHEREY-NAGEL GmbH & Co KG, Düren, Germany), following manual instructions.

### Enzyme Activity Assays and Biochemical Characterization

The enzymatic activity of recombinant ASPG was determined by the rate of NH_4_^+^ production during the hydrolysis of Asn. Aliquots of 1 μL of the purified protein were incubated during 30 min at 37°C in a volume of 15 μL activity buffer (50 mM HEPES pH 7.8, 50 mM KCl and 20 mM asparagine). The reactions were stopped by heating the samples for 2 min in a bath with boiling water followed by centrifugation at 12,400 ×*g* for 2 min. Free ammonia quantification was determined in the supernatant by the Berthelot phenol–hypochlorite method (Loan et al., 2013). After 30 min at room temperature absorbance at 635 nm was measured. Free ammonia was calculated using a standard curve obtained with different concentrations of ammonium sulfate. One unit of enzyme activity (1U) was defined as the amount of enzyme required to release 1 μmole of NH_4_^+^ per min.

To determine optimal temperature, samples were incubated in a Biometra Tgradient thermocycler (Biometra GmbH, Göttingen, Germany) with a 5°C interval temperature gradient ranging from 5 to 60°C. Enzymatic activity was determined by the rate of NH_4_^+^ production, as explained above. The activation energy was calculated by applying Arrhenius equation to the plotted data. The thermal stability was evaluated by incubating aliquots of the enzyme at temperatures ranging from 40 to 70°C without the presence of the Asn substrate, and determining enzymatic activity by the rate of NH_4_^+^ production. Relative activity for two different substrates (L-Asn and β-L-Asp-L-Ala) was determined by measuring the release of aspartate as described by Möllering (1985). Reactions were performed in microplates in a volume of 180 μL containing 50 mM HEPES pH 7.8, 50 mM KCl, 3.5 mM α-ketoglutarate, 0.6 mM NADH, 5 U each of glutamate-oxaloacetate transaminase (GOT) and malate dehydrogenase (MDH), and the substrate at the corresponding concentration. NADH oxidation was monitored using a “PowerWave HT Microplate Spectrophotometer” (Biotek Instruments, Winooski, VT, United States). To determine *K*_m_ and *V*_max_ values the assay based on measuring the release of aspartate described above was used. The concentrations of the substrates present in the reaction ranged from 0.2 to 10 times the value of *K*_m_. The experimental data were fitted to a Michaelis–Menten equation and the kinetic behavior was calculated by the Hanes–Woolf method (*r*^2^ > 0.99).

### Construction of Recombinant Vectors for PpASPG1 Expression in *E. coli*

Primers A1-13 and A1-14 were used to amplify the region spanning the ORF of *PpASPG1* or D3 variant using the plasmids described for *in planta* expression as templates. A secondary PCR reaction was performed with primers attB1 and attB2 for the introduction in Gateway vectors (Invitrogen Corporation, Carlsbad, CA, United States). The fragment was inserted in vector pDONR207 by recombination and transferred to the expression vector pDEST17. The expression vector was transferred to *E. coli* strain DH5α by electroporation for propagation and verification.

### Protein Expression in *E. coli* and Purification

The *E. coli* strain BL21-AI was transformed with the pDEST17. Protein expression was induced by adding 0.2% (w/v) arabinose.

Recombinant proteins were purified from *E. coli* inclusion bodies in denaturing conditions, applying Protino^®^ Ni-TED 2000 columns (MACHEREY-NAGEL GmbH & Co KG, Düren, Germany), following manual instructions. Protein samples were flash-frozen in liquid nitrogen and stored at -80°C. The electrophoretically separated proteins were visualized by silver stain following the recommendations of the “Silver stain Plus” kit (Bio-Rad, Hercules, CA, United States).

### MALDI-TOF Mass Spectrometry

The PpASPG1 purified protein was analyzed with a 4700 Proteomics Analyzer mass spectrometer (ABSCIEX) in linear mode to determine the molecular weight of the subunits. Subunits were also separated by SDS-PAGE and visualized by Coomassie colloidal staining. Spots were excised from the acrylamide gel and trypsin digested. Peptide mass fingerprinting (PMF) was acquired by working in the reflector positive ion mode. Peptide sequences were confirmed by TOF-TOF PSD (post source decay) fragmentation spectra.

### *In Silico* Analysis

Asparaginases amino acid sequences were obtained from following databases: https://phytozome.jgi.doe.gov/, http://arabidopsis.org/, http://congenie.org/ and http://www.scbi.uma.es/sustainpinedb/. A list of the different sequences with the corresponding ID and the database of provenance can be found in Supplementary Table S2. Sequence alignments and phylogenetic analysis were performed using the Mega6 software (Tamura et al., 2013).

## Results

**FIGURE 1:**
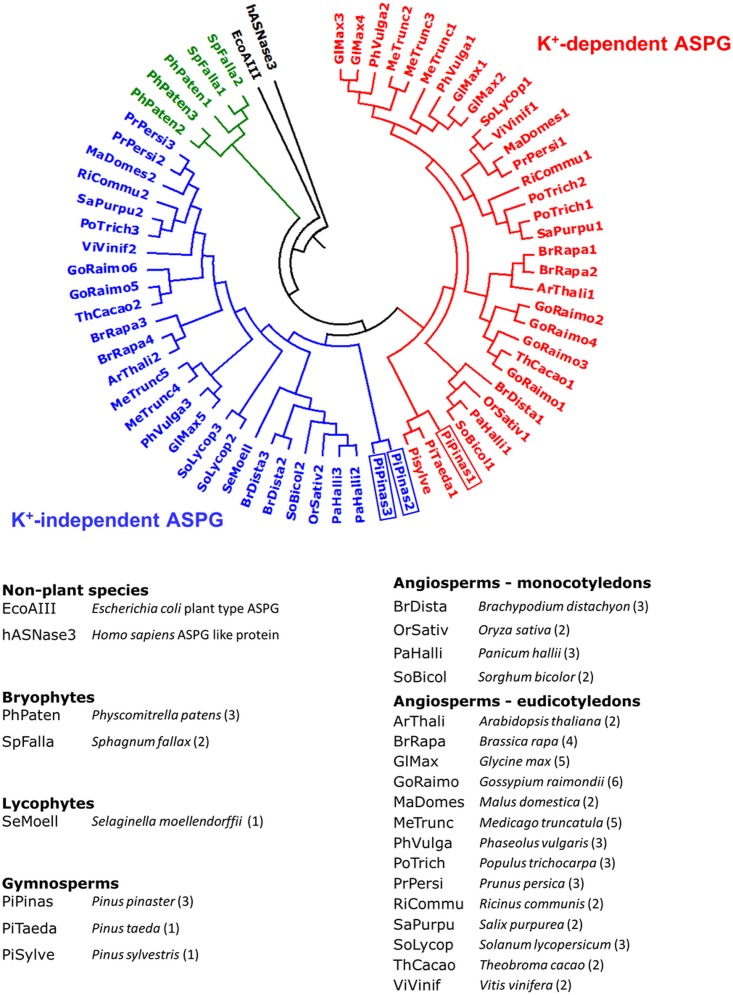
Circular dendrogram of plant-type L-asparaginases. Plant species whose genomes have been sequenced were explored for ASPG coding genes and entries with the highest similarity to pine ASPG were selected. The plant-type ASPG from *E. coli* and a human ASPG-like protein were included as outgroups. Clustering of the complete amino acid sequences was based on neighbor-joining analysis using MEGA version 6 software (Tamura et al., 2013). A bootstrap consensus tree was inferred from 1000 replicates. Red branches contain K^+^-dependent ASPG, blue branches include K^+^-independent ASPG. ASPG from bryophytes are marked in green. Below the phylogenetic tree the species included in the analysis and their corresponding abbreviations are listed. The number of different ASPG that were included for each species is mentioned in brackets. *P. pinaster* asparaginases are framed in rectangles in the dendrogram.

A search in the Sustainpine Database identified three different sequences including a full ORF encoding asparaginase in *P. pinaster* (sp_v3.0_unigene4029, sp_v3.0_unigene15077 and sp_v3.0_unigene36994), that were named *PpASPG1*, *PpASPG2* and *PpASPG3*, respectively. The phylogenetic analysis comparing the primary structure of the three proteins with plant ASPG of different species classified PpASPG1 as a K^+^-dependent asparaginase and PpASPG2 and PpASPG3 as K^+^-independent ASPG (**Figure 1**). The PpASPG1 sequence is 1396 nucleotides in size, including an ORF of 1128 nucleotides whereas both PpASPG2 and PpASPG3 sequences include a shorter ORF of 918 nucleotides. The difference in length of the ORFs is mainly the result of a higher number of codons encoding amino acids in the VL of the α-subunit C-terminus of PpASPG1 (Supplementary Figure S1).

The alignment in Supplementary Figure S1 includes plant ASPG that have been biochemically characterized and determined to be K^+^-dependent or K^+^-independent enzymes. The amino acids involved in anchoring the product in the active site and the catalytic switch are absolutely conserved in all the proteins in the alignment (Supplementary Figure S1), whereas those residues determining substrate specificity show some variability but with higher conservation among K^+^-dependent enzymes. According to Bejger et al. (2014), K^+^-dependence is linked to the VMDKSPHS motif (shaded in gray and labeled as activation in Supplementary Figure S1), and it was proposed that the last serine residue is involved in the potassium-dependence mechanism. However, the PpASPG1 protein has an apolar valine residue in the last position of this motif instead of a polar serine residue. This change is also present in the homolog proteins from *P. sylvestris* and *Pinus taeda*. As previously reported for *P. sylvestris* (Cañas et al., 2007), one significant difference between PpASPG1 and other K^+^-dependent ASPG in plants is the number of residues that compose the VL, which is exceptionally longer in the protein from *P. pinaster* and other pine species, such as *P. sylvestris* and *P. taeda*.

**FIGURE 2:**
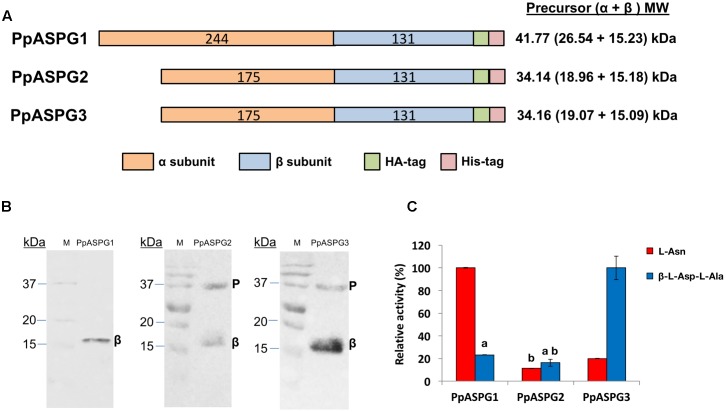
Expression of recombinant pine ASPG in *N. benthamiana* leaves and substrate preference. **(A)** Schematic representation of the structure of the three recombinant proteins that were expressed in tobacco leaves via agroinfection. The number of amino acids is included in the colored bars and the theoretical molecular mass corresponding to the precursor and subunits that would result from correct cleavage is indicated on the right. **(B)** Western blot analysis of tobacco leaf extracts expressing the recombinant proteins. Total soluble proteins (50 μg) from each leaf extract were separately analyzed by western blot with an antibody against the HA-tag. Bands marked with β correspond to molecular mass of the β subunit including the HA and His tags. Remaining uncleaved precursor (P) is visible in the PpASPG2 and PpASPG3 protein extracts. **(C)** Recombinant proteins were purified from the protein extracts and their relative activity was determined for two different substrates. For each substrate, catalytic activity is presented as the percentage of the highest specific activity measured at a substrate concentration of 25 mM. Values are the means of three independent replicates ± SD. Values labeled with the same letter are not significantly different according to *t*-tests (*p* < 0.01). The remainder values are significantly different.

The three cDNAs were transiently expressed in *N. benthamiana* leaves by agroinfection with the pJL-TRBO vector harboring each one of the three recombinant proteins as schematized in **Figure 2A**. All the three proteins were processed *in planta*, as indicated by the size of the peptide recognized by the anti-HA-tag antibody, which corresponds to the theoretical molecular mass for the processed β subunit (**Figure 2B**). Nevertheless, whereas PpASPG1 was fully processed, only part of the PpASPG2 and PpASPG3 precursors were processed, as shown by the remaining precursor band in the western blot analysis. The recombinant proteins produced in *N. benthamiana* were also purified and the specific activity of each protein was determined for two substrates, L-Asn and the isoaspartyl dipeptide β-L-Asp-L-Ala. PpASPG1 showed the highest activity for L-Asn, whereas the activities of PpASPG2 and PpASPG3 for this substrate were only 11 and 20% of the observed value for PpASPG1 (**Figure 2C**). PpASPG3 was the enzyme with the highest activity for the substrate β-L-Asp-L-Ala, and the activities of PpASPG1 and PpASPG2 were 23 and 16% of the activity observed for PpASPG3.

**FIGURE 3:**
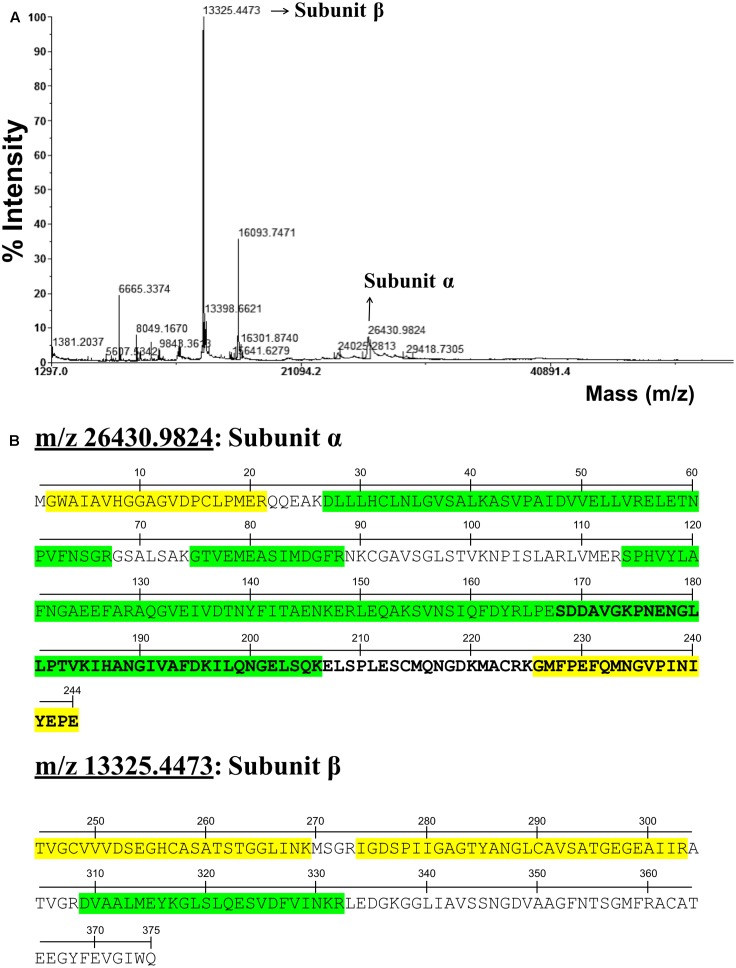
PpASPG1 protein analysis by mass spectrometry. **(A)** Spectrum obtained for undigested purified recombinant PpASPG1 protein by MALDI-TOF analysis in linear mode. **(B)** Identification of the peptides resulting from in gel digestion of the spots corresponding to m/z 26430.9824 and 13325.4473, marked upon the PpASPG1 protein sequence separated by its subunits. Green shaded letters: peptides identified by MALDI-TOF; yellow shaded letters: peptides that were fragmented and their sequences were confirmed by MS/MS; letters in bold: variable sequence region not conserved between homologous ASPG.

The exceptionally long VL of PpASPG1 together with the observation that the last 17 residues of the α subunit are removed from the mature recombinant EcAIII enzyme after the initial autolysis (Borek et al., 2004) prompted us to analyze the occurrence of the variable region in the mature enzyme. For pursuing this task with enough resolution to resolve the prevalence or elimination of amino acid residues in this region, we analyzed a purified preparation of recombinant and active protein by mass spectrometry. The molecular masses of both subunits were determined by MALDI-TOF mass spectrometry. As shown in **Figure 3A**, two peaks with masses of 26,431 and 13,325 were identified in the mass spectrum, quite close to the theoretical masses of the α and β subunits (26,535 and 13,327, respectively). Therefore, the experimental mass of the α subunit is slightly smaller than the theoretical mass. Both subunits were separated by SDS-PAGE and all bands with molecular mass in the range of 13 to 28 kDa were selected for trypsin digestion and analysis by MALDI TOF spectrometry in reflector mode, to ensure the identification of all possible variants of the processed α subunit. A total of 10 bands excised from the electrophoresis gel were analyzed. This analysis confirmed that the peptides of 26,431 and 13,325 were the α and β subunits of PpASPG1. The trypsin derived peptides from both subunits are shown in **Figure 3B**, which cover 76.6% of the α subunit and 60.3% of the β subunit. The results of mass profiles of the trypsin-derived peptides showed that the α subunit retains the complete VL in the mature protein. However, the N-terminal methionine residue is not present in the mature enzyme. Consequently, after the N-terminal methionine is removed, the theoretical molecular mass of the α subunit is 26,386 Da. The difference of 45 Da with the experimental molecular mass could be the result of a posttranslational modification. The occurrence of the complete VL in the mature PpASPG1 protein was further confirmed by analyzing the tryptic peptide from the C-end of the α subunit by MALDI TOF/TOF tandem mass spectrometry.

**FIGURE 4:**
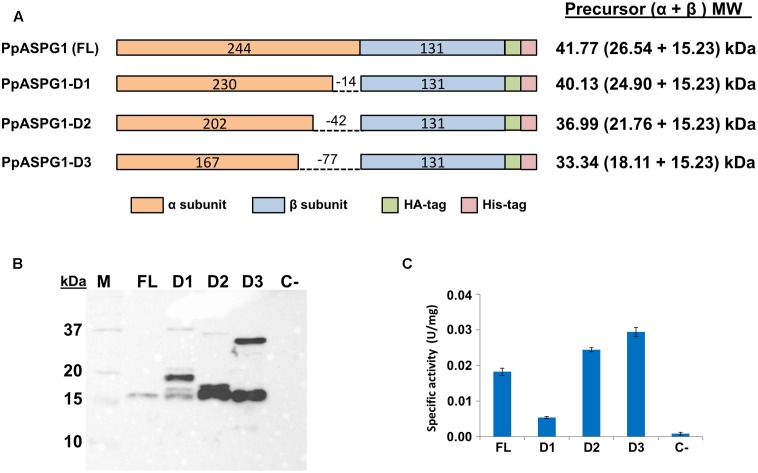
Production of PpASPG1 recombinant proteins in *N. benthamiana* with different deletions in the variable region. **(A)** Schematic representation of the different variants of PpASPG1 that were transiently expressed in tobacco leaves. Variants D1, D2, and D3 were expressed as a precursor from which several amino acids in the variable region were deleted, represented by a dashed line. The number of amino acids is included in the colored bars and the theoretical molecular mass corresponding to the precursor and subunits that would result from correct cleavage is indicated on the right. **(B)** Western blot analysis of the tobacco leaf extracts expressing the different versions of PpASPG1. An antibody against the HA-tag was used and 50 μg of proteins were loaded in each lane. Protein extract of a leaf that was infiltrated with the empty pJL-TRBO expression vector was also analyzed as a control (C-). **(C)** ASPG activity was determined in the protein extracts. Values are the means of three replicates ± SD. All values are significantly different according to *t*-tests (*p* < 0.01).

The role of this exceptionally long variable region at the C-end of the α subunit in the maturation and catalytic properties of the enzyme was analyzed by producing three variants of the recombinant PpASPG1 protein. Deletions in the ORF were generated to produce proteins lacking 14 (PpASPG1-D1), 42 (PpASPG1-D2), and 77 (PpASPG1-D3) residues from the C-terminal of the α subunit (**Figure 4A**). The full-length cDNA (FL) and those encoding mutant proteins were individually expressed in *N. benthamiana* leaves, and analyzed by western blot using an antibody against the HA-tag added at the C-end of the β subunit (**Figure 4B**). A polypeptide with a molecular mass corresponding to the β subunit was observed for all constructions, indicating that PpASPG1-D1, PpASPG1-D2, and PpASPG1-D3 were processed and the mature protein produced. This was further confirmed by detection of asparaginase activity in crude extracts, with detection of higher levels of asparaginase activity than in control leaves transformed with the empty pJL-TRBO vector (**Figure 4C**). However, in the crude extracts of leaves expressing any of the three modified proteins unprocessed precursor was detected whereas no precursor polypeptide was detected in crude extracts of leaves expressing the full PpASPG1 protein. The higher amount of unprocessed precursor was observed for PpASPG1-D3, which lacked the complete variable region. This observation was consistent in different replicas of the same experiment. Additionally, in crude extracts of leaves expressing PpASPG1-D1 and PpASPG1-D2 variants, β subunit polypeptides with abnormal higher molecular masses were observed. Since the HA-tag was added at the C-terminal of the β subunit, we hypothesize that those polypeptides are the result of incorrect processing of the precursor.

**FIGURE 5:**
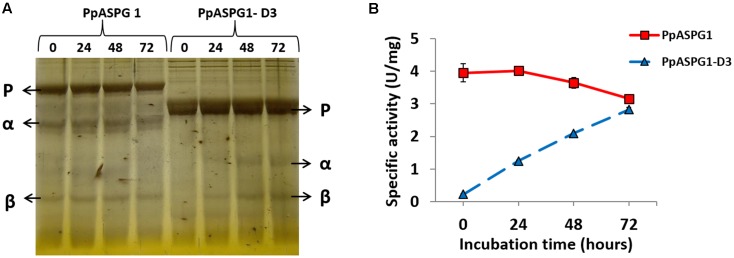
Elimination of the VL allows *in vitro* maturation of the PpASPG1 precursor. Both recombinant proteins were expressed in *E. coli* cells and recovered from inclusion bodies. The purified recombinant proteins were incubated at room temperature during three consecutive days in the presence of KCl 50 mM and aliquots were removed every 24 h and stored at –20°C until analysis. **(A)** Staining of the electrophoretically separated recombinant proteins. Each lane contains 5 μg of protein and the incubation time in hours is marked on top of the lane. **(B)** Remaining ASPG activity was measured in the 24-h interval aliquots for both proteins. Values are the means of three independent replicates ± SD. The specific activity values of PpASPG1 at 24, 48, and 72 h are not significantly different of the respective previous value according to *t*-tests (*p* < 0.01). The specific activity values of PpASPG1-D3 at 24, 48, and 72 h are significantly different of the respective previous value according to *t*-tests (*p* < 0.01).

To further characterize the influence of the variable region in the processing of PpASPG1 precursor we analyzed the ability of the recombinant protein produced in *E. coli* to undergo auto-processing *in vitro*. When both PpASPG1 and PpASPG1-D3 were expressed in *E. coli* and purified by using a His-tag added at the N-end of the precursor, most protein purified from inclusion bodies accumulated unprocessed (**Figure 5A**). Nevertheless, some processed α and β subunits were detected for PpASPG1 but not for the PpASPG1-D3 variant (**Figure 5A**, time 0). Increased levels of processed α and β subunits were observed for the PpASPG1-D3 variant after 24, 48, and 72 h of incubation at room temperature but no further processing was detected in the PpASPG1 protein (**Figure 5A**, time 24, 48, and 72). **Figure 5B** shows that protein processing was correlated with ASPG activity. At time 0, the activity of the D3 variant was almost negligible whereas for PpASPG1 it was approximately 4 U/mg. The specific activity did not increase with incubation time for PpASPG1, but gradually increased for PpASPG1-D3.

**FIGURE 6:**
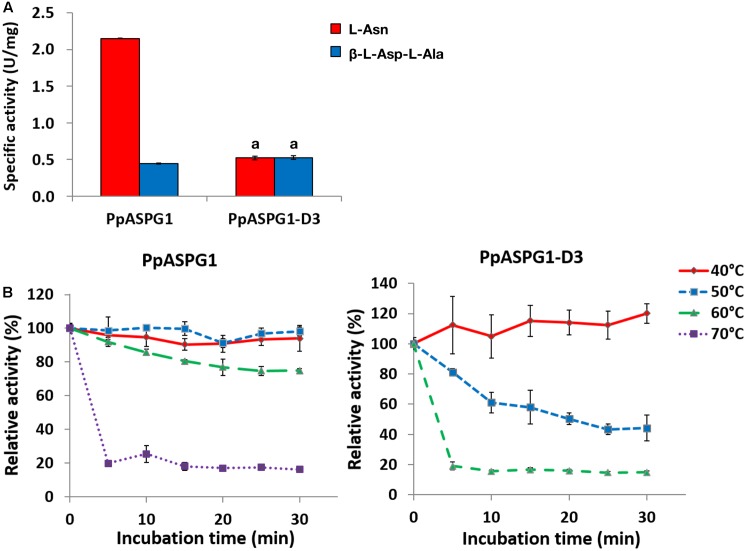
Effect of removing the VL on substrate preference and thermal stability of PpASPG1. Recombinant proteins PpASPG1 and PpASPG1-D3 (see **Figure 4A** for a schematic representation of the structure) were purified from agroinfiltrated tobacco leaves, and compared for substrate preference and thermal stability. **(A)** Catalytic activity was determined in a buffered medium containing 25 mM of the substrates asparagine or isoaspartyl-alanine dipeptide. Values are means of three replicates ± SD. Values labeled with the same letter are not significantly different according to *t*-tests (*p* < 0.01). The remainder values are significantly different. **(B)** The purified recombinant proteins were incubated at different temperatures. Aliquots were removed every 5 min and kept on ice until residual ASPG activity was measured. Values are means of three replicates ± SD. For PpASPG1, the residual activity values after incubating at 70°C are significantly different to those observed after incubating at 40°, 50, and 60°C according to *t*-tests (*p* < 0.01). The residual activity values after incubating at 50 or 60°C are significantly different only after incubating 25 and 30 min. The remainder values at the same incubation time are not significantly different. For PpASPG1-D3, all residual activity values after the same incubation time are significantly different according to *t*-tests (*p* < 0.01), except for values after incubating for 5 min at 40 and 50°C.

Functional and structural effects of removing the VL were evaluated by comparing substrate preference and thermal stability of PpASPG1 and PpASPG1-D3 (**Figure 6**). Removal of the VL caused a shift in the profile of substrate preference (**Figure 6A**) mainly as a result of changes in the *K*_m_ and *V*_max_ values for L-Asn and β-L-Asp-L-Ala (Supplementary Table S3). Consequently, the enzyme efficiency for L-Asn is dramatically reduced. No changes were observed in optimum pH with a value of 8, but the optimum temperature differed from 50°C for PpASPG1 to 35°C for PpASPG1-D3. The activation energy was 27.98 kJ/mol for PpASPG1 and 34.24 kJ/mol for PpASPG1-D3 (Supplementary Figure S2). Furthermore, removal of the VL affected the structural stability of the protein (**Figure 6B**), since the PpASPG1-D3 variant was clearly more susceptible to thermal inactivation. PpASPG1 activity was not affected after incubation 30 min at 50°C, and retained 75% of initial value after 30 min at 60°C. Conversely, PpASPG1-D3 activity was reduced to half of the starting value after 30 min at 50°C and very low activity was detectable after 30 min at 60°C.

**FIGURE 7:**
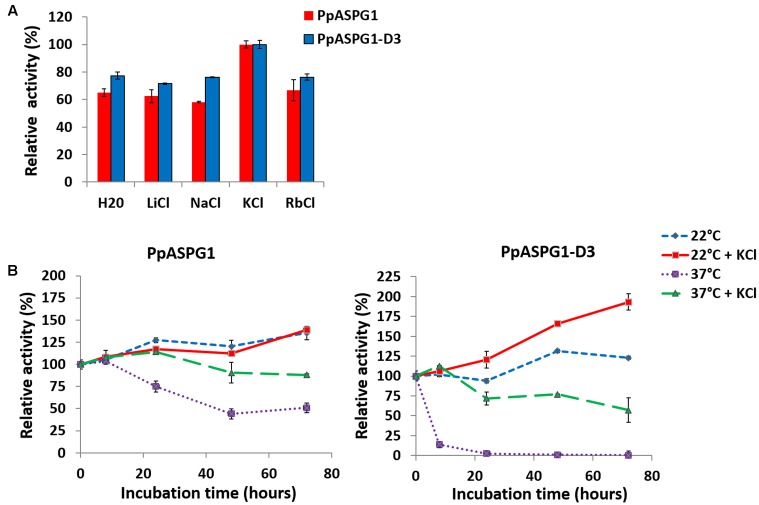
Effect of monovalent cations on enzymatic activity and thermal stability of both PpASPG1 variants. **(A)** ASPG activity was measured for PpASPG1 and PpASPG1-D3 in a buffered medium containing 20 mM L-Asn and 50 mM of the corresponding chloride salt for each ion or water. Values are presented as the mean of three replicates ± SD. For both PpASPG1 and PpASPG1-D3, the activity values in the presence of KCl are significantly different of those in the different conditions assayed, according to *t*-tests (*p* < 0.01). **(B)** Both protein variants were incubated during three consecutive days at 22 or 37°C in the buffered medium with or without the addition of 50 mM KCl. Aliquots were removed at different time points and residual ASPG activity was assayed. Values are presented as the mean of three replicates ± SD. For PpASPG1, in the absence of KCl, the activity values were significantly different between 22 and 37°C from the measurements after 24 h of incubation, according to *t*-test (*p* < 0.01). When KCl was present, the activity values are significantly different only at 48 and 72 h. At 22°C, there is not significantly differences in activity values at any incubation time between samples with or without KCl added. At 37°C activity values are significantly different between reactions with or without KCl added at 24, 48, and 72 h. For PpASPG1-D3, activity values are significantly different in the presence or absence of KCl at all incubation times both at 22 and 37°C, except at 8 h for 22°C, according to *t*-tests (*p* < 0.01). Similarly, activity values after incubation at 22 or 37°C are significantly different at all incubation times both in the absence or presence of KCl, except at 8 h with KCl.

Although, according to the primary structure, PpASPG1 is classified as a K^+^-dependent asparaginase, the substitution of serine for valine in the activation motif prompted us to analyze the effect of monovalent cations on the *P. pinaster* enzyme. Asparaginase activities for PpASPG1 and the PpASPG1-D3 variant were compared in the absence or presence of various monovalent cations. As shown in **Figure 7A**, the addition of Li^+^, Na^+^, and Rb^+^ cations did not affect the activity of both enzymes compared to the same reaction lacking any monovalent cation. However, a slight increase in activity was registered when K^+^ was added. We also determined the thermal stability of both proteins in the absence or presence of potassium ions (**Figure 7B**). The addition of K^+^ increased the thermal stability of both proteins, and the effect was more clearly contrasted in the case of the PpASPG1-D3, which has a lower stability to temperature. After 72 h of incubation at 37°C, no activity was detected for PpASPG1-D3 in the absence of the monovalent ion, whereas 62% of the initial activity was retained in the presence of K^+^ at 50 mM.

## Discussion

### The *P. pinaster* Transcriptome Contains At Least Three ASPG

Most of the ASPG that have been studied in plants belong to angiosperm species and little is known about these enzymes in gymnosperms or primitive terrestrial plants. Genomes of bryophytes include various *ASPG* genes and their sequences are approximately 57% identical to those of ASPG in vascular plants. However, our phylogenetic analysis (**Figure 1**) did not assign these sequences to either K^+^-dependent or K^+^-independent subgroups. In *Selaginella moellendorffii* only one *ASPG* gene was found which is more similar to the K^+^-independent ASPG. In databases of *Ceratopteris richardii*, *Ginkgo biloba*, and *Cycas rumphii* we did not find sequences with a significant similarity to pine ASPG probably due to low gene expression. Recently, the genomes of several conifer species have been published including *Picea glauca* (Birol et al., 2013; Warren et al., 2015), *P. abies* (Nystedt et al., 2013), and *P. taeda* (Neale et al., 2014; Zimin et al., 2014). Although the genome sequence for *P. pinaster* is not yet available, a reference transcriptome (Canales et al., 2014), contains three complete cDNA sequences for plant-type ASPG which can be classified as K^+^-dependent (*PpASPG1*) and -independent (*PpASPG2* and *PpASPG3)* according to their similarity with angiosperms sequences. Both *P. abies* and *P. taeda* genomes also contain three *ASPG* genes with high similarity to those of *P. pinaster*. Unfortunately, with the exception of one gene in *P. taeda* all sequences contained partial ORFs. Therefore, the phylogenetic tree (**Figure 1**) includes few gymnosperm entries, but the available information suggests that conifer genomes contain at least one gene coding for K^+^-dependent ASPG and two for the K^+^-independent type. Several angiosperm genomes have one *ASPG* gene of each subgroup and the phylogenetic tree also suggests events of genome duplications in some species that contain a higher number of genes. Our analysis based on conservation of primary structure suggests that the separation between subgroups of ASPG is related to spermatophytes, although genome sequences of more Pteridophyta and non-vascular species would be required in the analysis to confirm this hypothesis. No polyploidization events are known for any of the extant pine species (Morse et al., 2009) and whether the presence of two isoforms of the K^+^ independent type within conifers is related to a functional specialization remains to be investigated.

### All Three Pine ASPG Have Double Activity Asparaginase/Isoaspartyl Dipeptidase

Most ASPG that have been studied in angiosperms belong to the K^+^-independent type (Chang and Farnden, 1981; Lough et al., 1992; Casado et al., 1995; Hejazi et al., 2002; Borek et al., 2004). As far as we know K^+^-dependent ASPG have been characterized in only four species: *Pisum sativum* (Sodek and Lea, 1993), *A. thaliana* (Bruneau et al., 2006; Gabriel et al., 2012), *L. japonicus* (Credali et al., 2011) and *P. vulgaris* (Bejger et al., 2014). Different findings have been published about the biochemical characteristics of plant ASPG. Characterization of *Lupinus luteus* K^+^-independent ASPG and *E. coli* plant-type EcAIII suggested a main function as isoaspartyl peptidases, while Asn hydrolysis is of secondary importance (Borek et al., 2004). Similar results were reported for the human plant-type asparaginase (hASNase3), which also presents ASPG activity but has more affinity toward isoaspartyl dipeptides (Cantor et al., 2009). The first characterizations of both subtypes within the same species in angiosperms indicated that only the K^+^-independent ASPG has a double catalytic activity while K^+^-dependent ASPG only hydrolyzes Asn (Bruneau et al., 2006; Credali et al., 2011). However, later analysis of *A. thaliana* enzymes showed that the K^+^-dependent ASPG also has hydrolytic activity for isoaspartyl dipeptides (Gabriel et al., 2012), which is in agreement with our observations with the pine enzyme.

Analysis of the recombinant pine ASPG proteins produced in *N. benthamiana* indicates that the three enzymes are able to hydrolyze both substrates, Asn and isoaspartyl dipeptides (**Figure 2C**), but with differential substrate preference. PpASPG3 shows a clear preference for isoaspartyl alanine with a higher *V*_max_ as well as a higher affinity for this substrate (Supplementary Table S3); it catalyzes β-L-Asp-L-Ala hydrolysis almost 22 times more efficiently than Asn hydrolysis. Its characteristics are comparable with those described for K^+^-independent ASPG, indicating that its main function is related to degradation of proteins with β-L-isoaspartyl peptide bonds. In *L. japonicus*, the K^+^-independent ASPG had a *K*_m_ of 30 mM for Asn and 0.54 mM for β-Asp-His (Credali et al., 2011) and the orthologous ASPG in *L. luteus* exhibits a *K*_m_ value of 4.8 mM for Asn and 0.14 mM for β-Asp-Leu (Borek et al., 2004). For PpASPG2 the *V*_max_ values for both substrates were similar and very low so that the collected data could not be fitted to a Michaelis–Menten model. However, we could not rule out that the preferred substrate is an isoaspartyl dipeptide different to β-L-Asp-L-Ala. PpASPG1 was almost 19 times more efficient with Asn than with isoaspartyl alanine, confirming that Asn hydrolysis is the main function of this enzyme, as described for its orthologs enzymes in angiosperms. However, our data also show that PpASPG1 is able to catalyze less efficiently the hydrolysis of isoaspartyl dipeptides, as shown for the product of At3g16150 gene (Gabriel et al., 2012).

### The Mature PpASPG1 Enzyme Retains the Complete Variable Region

Plant ASPG, as Ntn-hydrolase family members, are expressed as precursors that undergo a proteolytic activation process placing the catalytic nucleophile residue at the N-terminal of the β subunit. In PpASPG1 and other conifer ASPG, the VL is remarkably long and spans 77 amino acids for which we did not find any sequence similarity in ASPG of non-conifer eukaryotes, except for the last 13 amino acids preceding the nucleophile. Results of peptide fingerprinting and MS/MS show that the complete sequence of the α subunit, including the VL, is present in the mature active enzyme, except for Met1 (**Figure 3B**). In *L. japonicas*, the VL of ASPG was also present in the mature enzyme (Credali et al., 2011) and it is not unusual for eukaryote expressed proteins to have the N-terminal methionine eliminated (Meinnel et al., 2005). In addition, the m/z analysis indicated a possible sulfoxidation of Met in positions 19, 79, 84, 227, and 233 and oxidation of Trp in position 3. These posttranslational modifications may account for the differences between the m/z of the peptides and their theoretically calculated molecular weight.

When comparing the length of the sequence, some ASPG of bryophytes (PhPaten2 and PhPaten3) also have a long region of approximately 60 residues, but the sequence does not share similarity with that of PpASPG1. The longer VL sequence in the pine enzyme does not seem to have an impact on the mature enzyme, since its function is similar to that of ASPG in angiosperms with a shorter VL. Nevertheless, conservation of VL in conifers may have a role or beneficial effect related to maturation, structure, or enzyme activity. For this reason, we compared the biochemical characteristics of PpASPG1 and mutant proteins with deletions in the VL.

### Effects of VL on Maturation of PpASPG1

The activation of plant ASPG has been addressed by various authors and described as an autoproteolytic maturation process in *A. thaliana* (Bruneau et al., 2006; Gabriel et al., 2012), *L. luteus* (Michalska et al., 2006), *L. japonicus* (Credali et al., 2011), and *P. vulgaris* (Bejger et al., 2014). In contrast, the ASPG from *P. sylvestris* expressed in *E. coli* did not show autocatalytic activity *in vitro* (Cañas et al., 2007). Because of the lack of electron density at the C-terminal of the α subunit of crystallized K^+^-independent ASPG from *L. luteus* (Michalska et al., 2006) and K^+^-dependent ASPG from *P. vulgaris* (Bejger et al., 2014), the role of the VL sequence in the process of autocatalytic activation, substrate binding and catalysis and K^+^ activation cannot be evaluated by protein modeling. Therefore, we decided to compare *in planta* the autoproteolysis efficiency of four variants of precursors containing gradually shorter VL. Deletions were performed keeping glutamate as the last α-subunit residue hence the scissile bond to be cleaved for activation in each case was Glu(x)-Thr245. When the different recombinant PpASPG1 variants were expressed in *N. benthamiana* leaves only the unmodified protein was completely processed, although none of the mutations caused total suppression of the activation process. Furthermore, these protein variants produced some aberrantly processed peptides. These results suggest that the full sequence of VL is required for an efficient proteolysis and correct positioning of the activation cleavage.

It is generally observed that, when expressed in *E. coli* cells, the autoproteolysis of plant ASPG is a slow process that continues *in vitro* (Michalska and Jaskólski, 2006; Cantor et al., 2009; Nomme et al., 2012). To evaluate the autoproteolytic capacity of conifer K^+^-dependent ASPG, PpASPG1, and PpASPG1-D3 were expressed in *E. coli*, and both proteins were recovered and purified from inclusion bodies, primarily in their inactive unprocessed precursor form. However, a certain amount of protein processed *in vivo* was observed in the case of PpASPG1, whereas in the case of PpASPG1-D3 this was not detected, which agrees with the higher processing efficiency of PpASPG1 observed when both proteins were produced *in planta*. After 3 days of incubation at room temperature, no signs of *in vitro* cleavage were detected for PpASPG1 whereas the PpASPG1-D3 form did show autoproteolytic activity. Our results indicate that maturation of PpASPG1 might require the assistance of another protein or certain specific conditions as suggested by Cañas et al. (2007) and apparently the VL poses an obstacle for the autocatalytic activation *in vitro*. Because the sequence flanking the nucleophile is not conserved between Ntn-hydrolases, there may be some differences in the particular autoproteolytic process between orthologs (Michalska et al., 2008; Nomme et al., 2012; Su et al., 2013). The VL of angiosperm ASPG is supposed to be unstructured (Bejger et al., 2014), but no information is available for the sequence that is unique to conifer K^+^-dependent ASPG. Structure analysis by crystallization would provide more insight about the autocatalytic activation of ASPG1 and more experiments are required to determine if the VL could provide a posttranslational control mechanism for enzyme activation in conifers.

### The VL Influences Some Biochemical Characteristics of PpASPG1

It has been suggested that the VL might be involved in substrate preference of plant ASPG. In *Arabidopsis*, chimeric proteins with the VL of K^+^-dependent ASPG exchanged for the loop of its K^+^-independent isoform had reduced autoproteolytic capacity and *V*_max_ but the *K*_m_ value for Asn and isoaspartyl dipeptides was hardly affected. The reciprocal recombinant protein carrying the K^+^-dependent loop in the K^+^-independent backbone had little effect on autoproteolysis but increased the efficiency of Asn hydrolysis by 60-fold (Gabriel et al., 2012). PpASPG1 clearly showed a higher preference for Asn as a substrate than for β-L-Asp-L-Ala by almost 19-fold (**Figure 2C** and Supplementary Table S3). However, elimination of VL in PpASPG1-D3 determined that this enzyme hydrolyzed Asn and isoaspartyl alanine with almost equal efficiency. In addition, *V*_max_ of the modified protein was only about 1/4 of the value for PpASPG1, but this could be because of a large quantity of the purified recombinant protein that remained inactive in its unprocessed precursor form. This suggests that the VL largely reduces the binding capacity of β-L-Asp-L-Ala in the catalytic center in contrast to the smaller Asn molecule, or possibly it hinders the entrance to the active site cavity as was suggested for ASPG from yellow lupin (Michalska et al., 2006).

The substantial differences observed in the optimum temperature and activation energy for PpASPG1 and PpASPG1-D3 suggested that the VL somehow protects the enzyme against thermal degradation and reduces the activation energy. In fact, incubation at different temperatures showed that PpASPG1 enzyme is more stable than PpASPG1-D3 (**Figure 6B**). The length of these linker sequences has evolved from short in bacteria and Archaea to long in eukaryotes as genome complexity increases, although plants generally have the shortest linker sequences within eukaryote organisms (Wang et al., 2011). Possibly the long VL of PpASPG1 could imply advantages in the stability and folding of the enzyme.

### Enhancement of Activity by Potassium Cations in PpASPG1 Is Limited

PpASPG1 has a higher similarity to the K^+^-dependent type ASPG in angiosperms (**Figure 1**). Both, PpASPG1 and PpASPG1-D3 were most active when K^+^ was included in the buffer (**Figure 7A**), which would validate the classification of PpASPG1 within the K^+^-dependent group of ASPG. However, in the presence of other ions or water they maintained 65% to 75% of their catalytic activity, which makes them far less dependent of K^+^ than their orthologs in angiosperms which suffer a 90% loss of activity in absence of the cation (Bruneau et al., 2006; Credali et al., 2011). The three amino acids that were referred to as the catalytic switch (His117, Arg224, and Glu250 in the PvAspG1 protein) by Bejger et al. (2014) are conserved amongst all sequences included in the alignment of Supplementary Figure S1 and also among all the sequences included in the phylogenetic three (**Figure 1**). Nevertheless, it is the binding of the metal ion in the activation loop what induces the conformational changes in these residues setting this catalytic switch to ON or OFF to allow or prevent anchoring of the reaction substrate/product in the active site (Bejger et al., 2014). Therefore, the differential effect of K^+^ ion on the conifer enzyme may be as a result of differences in the sequence of the activation loop. In the PvAspG1 protein the activation loop is formed by eight residues (VMDKSPHS) (see Supplementary Figure S1). In particular, Ser118 seems to be of key importance for the potassium dependence of PvAspG1 and it is substituted in PpASPG1 and in the independent K^+^-enzymes by a hydrophobic amino acid (valine or isoleucine). This residue is responsible for the proper positioning of water molecules coordinated by the metal ion and also mediates the conformational changes of Glu250, one of the catalytic switch residues, effected by the exchange of the coordinated alkali-metal cation (Bejger et al., 2014). In fact, this serine residue is absolutely conserved in all K^+^-dependent proteins in the phylogenetic tree on **Figure 1** with the exception of the conifer protein. Consequently, this difference in the activation loop of the pine enzyme may prevent the proper functioning of the ON/OFF K^+^-dependent mechanism described for its orthologs in angiosperms.

Since PpASPG1-D3 only differs from the PpASPG1 complete sequence in lacking the VL and the activation loop is kept intact, the effect of the metal ion was expected to be similar for both proteins. We found a larger difference in relative activity with/without K^+^ for PpASPG1 than for PpASPG1-D3, but this could be due to the higher stability of the enzyme carrying the VL. Incubation of the two recombinant ASPG during 3 days in the presence and absence of K^+^ suggests that the metal ion contributes in maintaining the functional integrity of the enzyme (**Figure 7B**) as also reported for pea ASPG (Sodek, 1980). In fact, this protective effect against denaturalization might explain the higher activity of the PpASPG1 in the presence of K^+^ instead of an actual activation mechanism as described in angiosperms.

## Author Contributions

FMC and FRC designed the experiments, FdT, RC, and CA contributed in the design of some experiments. SVK and FdT performed the experiments. SVK, FMC, and FRC analyzed the data and wrote the manuscript. All authors have read and approved the final manuscript.

## Conflict of Interest Statement

The authors declare that the research was conducted in the absence of any commercial or financial relationships that could be construed as a potential conflict of interest.
