# Supplementary material for: Characterization of Three L-Asparaginases from Maritime Pine (Pinus pinaster Ait.)
Source: Front Plant Sci. 2017 Jun 23;8:1075. doi: 10.3389/fpls.2017.01075 (PMC5481357; doi:10.3389/fpls.2017.01075)
Supplement: Supplementary file 1 [file Table_1.PDF]

Supplementary Table S1. Primer sequences.

Primer reference	Primer sequence
A1-1	GGGTGCATCCTACTGCCAAAT
A1-2	GCCACCCTTACCATCTTCCAA
A1-3	GTGGTTGTTCTGTGGGAAGA
A1-4	CTAGTTAATTAACATGGGATGGGCCATAGCT
A1-5	AGAGGCGGCCGCCTACTGCCAAATGCC
A1-6	GGAATGTTCCCGGAGACTGTGGGTTGTGTTG
A1-7	CAACACAACCCACAGTCTCCGGGAACATTCC
A1-8	CCTTCAGAATGGGGAAACTGTGGGTTGTGTTG
A1-9	CAACACAACCCACAGTTTCCCCATTCTGAAGG
A1-10	GTTTGACTACCGTCTCCCAGAAACTGTGGGTTGTGTTG
A1-11	CAACACAACCCACAGTTTCTGGGAGACGGTAGTCAAAC
A1-12	CATCGCGGCCGCTTAGTGATGGTGATGGTGATGCGCATAGTCAGGAACATCGTATGGGTACTGCCAAATGCCTACTTC
A1-13	AAAAAGCAGGCTTCATGGGATGGGCCATAGCT
A1-14	AGAAAGCTGGGTCCTACTGCCAAATGCCTACTTCAAA
A2-1	GGAAGGCGAGAGGCAG
A2-2	GCATACCACATAACAATCCCAAG
A2-3	GAGGTTAATTAACATGGGGTGGGCAATTGCCTTG
A2-4	AGAAGCGGCCGCTTAGTGATGGTGATGGTGATGCGCATAGTCAGGAACATCGTATGGGTATGCCAATATTCCCACTTC
A3-1	GAAGGGGAGGGACGAG
A3-2	ATGCATGTTAAGACATCAGTT
A3-3	GAGCTTAATTAACATGGGGTGGGCACTTGCGTTG
A3-4	CAGTGCGGCCGCTTAGTGATGGTGATGGTGATGCGCATAGTCAGGAACATCGTATGGGTATGCCAATATTCCCACTTC
attB1	GGGGACAAGTTTGTACAAAAAAGCAGGCT
attB2	GGGGACCACTTTGTACAAGAAAGCTGGGT
