# Supplementary material for: Characterization of Three L-Asparaginases from Maritime Pine (Pinus pinaster Ait.)
Source: Front Plant Sci. 2017 Jun 23;8:1075. doi: 10.3389/fpls.2017.01075 (PMC5481357; doi:10.3389/fpls.2017.01075)
Supplement: Supplementary file 2 [file Table_2.PDF]

Supplementary Table S2. List of sequences used in the phylogenetic analysis.

Tree ID.	Species	Database	Sequence ID
**Non-plant species**			
EcoAIII	*Escherichia coli*	GenBank	EFO58337
hASNase3	*Homo sapiens*	GenBank	AAM28434
**Bryophytes**			
PhPaten1	*Physcomitrella patens*	Phytozome 11.0 (JGI)	Pp3c14_14930
PhPaten2	*Physcomitrella patens*	Phytozome 11.0 (JGI)	Pp3c8_21770
PhPaten3	*Physcomitrella patens*	Phytozome 11.0 (JGI)	Pp3c23_6900
SpFalla1	*Sphagnum fallax*	Phytozome 11.0 (JGI)	Sphfalx0119s0066
SpFalla2	*Sphagnum fallax*	Phytozome 11.0 (JGI)	Sphfalx0119s0065
**Lycophytes**			
SeMoell	*Selaginella moellendorffii*	UniProtKB	D8T3N6
**Gymnosperms**			
PiPinas1	*Pinus pinaster*	Sustainpine V.3.0	unigene4029
PiPinas2	*Pinus pinaster*	Sustainpine V.3.0	unigene15077
PiPinas3	*Pinus pinaster*	Sustainpine V.3.0	unigene36994
PiSylve	*Pinus sylvestris*	GenBank	CAK22360
PiTaeda	*Pinus taeda*	Congenie.org	PITA_000017050
**Angiosperms - monocotyledons**			
BrDista1	*Brachypodium distachyon*	Phytozome 11.0 (JGI)	Bradi5g24227
BrDista2	*Brachypodium distachyon*	Phytozome 11.0 (JGI)	Bradi1g15126
BrDista3	*Brachypodium distachyon*	Phytozome 11.0 (JGI)	Bradi1g15130
OrSativ1	*Oryza sativa*	Phytozome 11.0 (JGI)	LOC_Os04g55710
OrSativ2	*Oryza sativa*	Phytozome 11.0 (JGI)	LOC_Os03g40070
PaHalli1	*Panicum hallii*	Phytozome 11.0 (JGI)	Pahal.G02572
PaHalli2	*Panicum hallii*	Phytozome 11.0 (JGI)	Pahal.I02619
PaHalli3	*Panicum hallii*	Phytozome 11.0 (JGI)	Pahal.I02618
SoBicol1	*Sorghum bicolor*	Phytozome 11.0 (JGI)	Sobic.006G243200
SoBicol2	*Sorghum bicolor*	Phytozome 11.0 (JGI)	Sobic.001G174700
**Angiosperms - eudicotyledons**			
ArThali1	*Arabidopsis thaliana*	TAIR (www.arabidopsis.org)	AT3G16150
ArThali2	*Arabidopsis thaliana*	TAIR (www.arabidopsis.org)	AT5G08100
BrRapa1	*Brassica rapa*	Phytozome 11.0 (JGI)	Brara.A03076
BrRapa2	*Brassica rapa*	Phytozome 11.0 (JGI)	Brara.E02537
BrRapa3	*Brassica rapa*	Phytozome 11.0 (JGI)	Brara.J02454
BrRapa4	*Brassica rapa*	Phytozome 11.0 (JGI)	Brara.J02455
GlMax1	*Glycine max*	UniProtKB	I1MXK5
GlMax2	*Glycine max*	UniProtKB	I1M8R0
GlMax3	*Glycine max*	UniProtKB	I1K824
GlMax4	*Glycine max*	UniProtKB	I1JTM6
GlMax5	*Glycine max*	UniProtKB	C6TER3
GoRaimo1	*Gossypium raimondii*	Phytozome 11.0 (JGI)	Gorai.005G101000
GoRaimo2	*Gossypium raimondii*	Phytozome 11.0 (JGI)	Gorai.009G276200
GoRaimo3	*Gossypium raimondii*	Phytozome 11.0 (JGI)	Gorai.010G023000
GoRaimo4	*Gossypium raimondii*	Phytozome 11.0 (JGI)	Gorai.009G215700
GoRaimo5	*Gossypium raimondii*	Phytozome 11.0 (JGI)	Gorai.008G200400
GoRaimo6	*Gossypium raimondii*	Phytozome 11.0 (JGI)	Gorai.007G040100
MaDomes1	*Malus domestica*	Phytozome 11.0 (JGI)	MDP0000263180
MaDomes2	*Malus domestica*	Phytozome 11.0 (JGI)	MDP0000658649
MeTrunc1	*Medicago truncatula*	UniProtKB	Q2HTR7
MeTrunc2	*Medicago truncatula*	UniProtKB	G7I4Z5
MeTrunc3	*Medicago truncatula*	UniProtKB	G7J705
MeTrunc4	*Medicago truncatula*	UniProtKB	A0A072URR5
MeTrunc5	*Medicago truncatula*	UniProtKB	A0A072USJ6
PhVulga1	*Phaseolus vulgaris*	UniProtKB	V7CU13
PhVulga2	*Phaseolus vulgaris*	UniProtKB	V7AST5
PhVulga3	*Phaseolus vulgaris*	UniProtKB	V7CAP3
PoTrich1	*Populus trichocarpa*	Phytozome 11.0 (JGI)	Potri.014G022900
PoTrich2	*Populus trichocarpa*	Phytozome 11.0 (JGI)	Potri.002G122900
PoTrich3	*Populus trichocarpa*	Phytozome 11.0 (JGI)	Potri.012G063400
PrPersi1	*Prunus persica*	UniProtKB	M5XEH2
PrPersi2	*Prunus persica*	UniProtKB	M5WUV5
PrPersi3	*Prunus persica*	Phytozome 11.0 (JGI)	Prupe.5G209200
RiCommu1	*Ricinus communis*	Phytozome 11.0 (JGI)	30170.t000233
RiCommu2	*Ricinus communis*	Phytozome 11.0 (JGI)	30138.t000099
SaPurpu1	*Salix purpurea*	Phytozome 11.0 (JGI)	SapurV1A.0919s0120
SaPurpu2	*Salix purpurea*	Phytozome 11.0 (JGI)	SapurV1A.0789s0060
SoLycop1	*Solanum lycopersicum*	UniProtKB	K4BUT1
SoLycop2	*Solanum lycopersicum*	UniProtKB	K4C8B3
SoLycop3	*Solanum lycopersicum*	UniProtKB	K4BKY1
ThCacao1	*Theobroma cacao*	UniProtKB	A0A061FBS8
ThCacao2	*Theobroma cacao*	UniProtKB	A0A061FX99
ViVinif1	*Vitis vinífera*	Phytozome 11.0 (JGI)	GSVIVG01009430001
ViVinif2	*Vitis vinifera*	Phytozome 11.0 (JGI)	GSVIVG01008150001
