# Supplementary material for: Characterization of Three L-Asparaginases from Maritime Pine (Pinus pinaster Ait.)
Source: Front Plant Sci. 2017 Jun 23;8:1075. doi: 10.3389/fpls.2017.01075 (PMC5481357; doi:10.3389/fpls.2017.01075)
Supplement: Supplementary file 3 [file Table_3.PDF]

Supplementary Table S3. Kinetic parameters of pine asparaginases and the mutant form.

	Substrate	K_m (mM)	V_{max} (U/mg)	V_{max}/K_m (U/mg.mM)
**PpASPG1**	L-Asn	4.58 ± 0.03	2.59 ± 0.01	0.56
	β-Asp-Ala	56.73 ± 13.88 *	1.46 ± 0.28 *	0.03
**PpASPG2**	L-Asn	ND	0.27 ± 0.01	ND
	β-Asp-Ala	ND	0.37 ± 0.05	ND
**PpASPG3**	L-Asn	6.12 ± 0.96	0.53 ± 0.01	0.09
	β-Asp-Ala	1.04 ± 0.21	2.03 ± 0.23	1.96
**PpASPG1-D3**	L-Asn	7.16 ± 0.88	0.68 ± 0.05	0.09
	β-Asp-Ala	9.44 ± 0.82	0.72 ± 0.05	0.08

*apparent value.

ND: K_m could not be determined due to low enzymatic activity.
