# Supplementary material for: Characterization of Three L-Asparaginases from Maritime Pine (Pinus pinaster Ait.)
Source: Front Plant Sci. 2017 Jun 23;8:1075. doi: 10.3389/fpls.2017.01075 (PMC5481357; doi:10.3389/fpls.2017.01075)
Supplement: Supplementary file 4 [file Image_1.PDF]

```
                        Sub.spec.
PpASPG1     -MGWAIAVHGGAGVD-PCLPMERQQEAKDLLLHCLNLGVSALKASVPAIDVVELLVRELE   58
At3g16150   MGGWAIAVHGGAGID-PNLPAERQEEAKQLLTRCLNLGIIALRSNVSAIDVVELVIRELE   59
PvAspG1     MGGWAIAVHGGAGVD-PTLPLERQEEAKQLLTRCLNLGISALNSNVPAIDVVELVVRELE   59
LjNSE1      MGRWAIAVHGGAGVD-PNLPLQRQEEAKQLLTRCLNLGISALTSNLSAIDVVELVVRELE   59
At5g08100   MVGWAIALHGGAGDIPIDLPDERRIPRESALRHCLDLGISALKSGKPPLDVAELVVRELE   60
PpASPG2     -MGWAIALHGGAGDIARTVTSERSERAEAGIRHCLQIGVAALRESRSALDVAEIVVRELE   59
PpASPG3     -MGWALALHGGARDIARTVTPERREIAEVDLRHCLQIGVAALKESRTALDVAEIVVRELE   59
               **:*:****      :  :*    :  : :**::*: **      :**.*:::****

                                                             Activation
PpASPG1     TNPVFNSGRGSALSAKGTVEMEASIMDGFRNKCGAVSGLSTVKNPISLARLVMERSPHVY   118
At3g16150   TDPLFNSGRGSALTEKGTVEMEASIMDGTKRRCGAVSGITTVKNPISLARLVMDKSPHSY   119
PvAspG1     TDPLFNSGRGSALTEKGTVEMEASIMDGPKRRCGAVSGLTTVKNPISLARLVMDKSPHSY   119
LjNSE1      TDPIFNSGRGSALTEKGTVEMEASIMDGPKRRCGAVSGVTTVKNPISLARLVMDKSPHSY   119
At5g08100   NHPDFNAGKGSVLTAQGTVEMEASIMDGKTKRCGAVSGLTTVVNPISLARLVMEKTPHIY   120
PpASPG2     NDPTFNAGRGSVLTEVGTVEMEATIMDGPTKHCGAVSGLSTVVNPISLARLVMEKTPHIY   119
PpASPG3     NNPSFNAGIGSVLTEEGTIEMEASIMEGSTKRCGAVSGISTVVNPISLARLVMEKTPHIY   119
            ..* **:* **.*:  **:****:**:*  .:******::** **********:::** *

PpASPG1     LAFNGAEEFARAQGVEIVDTNYFITAENKERLEQAKSVNSIQFDYRLPESDDAVGKPNEN   178
At3g16150   LAFSGAEDFARKQGVEIVDNEYFVTDDNVGMLKLAKEANSILFDYRIPPMGCA-------   172
PvAspG1     IAFSGAEDFARQQGVEVVDNEYFVTPDNVGMLKLAKEANTILFDYRIPSSAYE-------   172
LjNSE1      LAFDGAEDFARKQGVELVDNEYFITPDNVGMLKLAKEAKAILFDYRVPTTAYE-------   172
At5g08100   LAFDAAEAFARAHGVETVDSSHFITPENIARLKQAKEFNRVQLDYTVPSPKVPD------   174
PpASPG2     LAFDGAEKFAREQGVDTTDAKHFITEENVERLRRARATHTVQVDFSTPTESI--------   171
PpASPG3     LAFDGAEKFAREQGVDTTDTKYFITKEIVEQLQQVRETQAVQVAFSAPTESN--------   171
            :**..** *** :**: .: .:*:* :    *. .:  . : . :  *

PpASPG1     GLLPTVKIHANGIVAFDKILQNGELSQKELSPLESCMQNGDKMACRKGMFPEFQMNGVPI   238
At3g16150   -------------------------------------------GA-AATDSPIQMNGLPI   188
PvAspG1     -------------------------------------------TCGSGVESPLQMNGLPI   189
LjNSE1      -------------------------------------------TCGAGVESPLYMNGMPI   189
At5g08100   -------------------------------------------NC---------------   176
PpASPG2     ------------------------------------------------------------   171
PpASPG3     ------------------------------------------------------------   171

                  ↓                           D  D                   D D
PpASPG1     NIYEPETVGCVVVDSEGHCASATSTGGLINKMSGRIGDSPIIGAGTYANGLCAVSATGEG   298
At3g16150   SIYAPETVGCVVVDGKGHCAAGTSTGGLMNKMMGRIGDSPLIGAGTYASEFCGVSCTGEG   248
PvAspG1     SVYAPETVGCVVVDREGRCAAATSTGGLMNKMTGRIGDSPLIGAGTYACDVCGVSCTGEG   249
LjNSE1      SVYAPETVGCVVVDREGRCAAATSTGGLMNKMTGRIGDSPLIGAGTYACDVCGVSCTGEG   249
At5g08100   GDSQIGTVGCVAVDSAGNLASATSTGGYVNKMVGRIGDTPVIGAGTYANHLCAISATGKG   236
PpASPG2     --CHQETVGCVVVDSLGNCVAATSTGGLVNKMVGRIGDTPVVGAGNYANHLCAVSATGRG   229
PpASPG3     --SHPETVGCAVVDSLGNCVAATSTGGLMNKMVGRIGDTPIIGAGTYANHLCAVSATGMG   229
                  ****..**  *. .:.***** :*** *****:*::***.**  .*.:*.** *
                                               Sub.spec
PpASPG1     EAIIRATVGRDVAALMEYKGLSLQESVDFVINKRLEDGKGGLIAVSSNGDVAAGFNTSGM   358
At3g16150   EAIIRATLARDVSAVMEYKGLNLQEAVDYVIKHRLDEGFAGLIAVSNKGEVVCGFNSNGM   308
PvAspG1     EAIIRGTLAREVAAVMEYKGLKLHQAVDFVIKHRLDEGKAGLIAVSNTGEVACGFNCNGM   309
LjNSE1      EAIIRGTLAREVAAVMEYKGLGLQQAVDFVIKHRLDEGLAGLIAVSHTGEVAYGFNCNGM   309
At5g08100   EDIIRGTVARDVAALMEYKGLSLTEAAAYVVDQSVPRGSCGLVAVSANGEVTMPFNTTGM   296
PpASPG2     EYIIRATVARDVAALMEYKGFSLKEAANYVIG-HMEKGTCGLIAVSSNGEVVMPFNTSGM   288
PpASPG3     EHIIQATVARDVAALMEYKGFSVKEAANYVMG-NMEKGTCGLVAVSCNGEVAMPFNTSGM   288
            * **:.*:.*:*:*:*****: : ::. :*:   :  *  **:*** .*:*.  ** .**

PpASPG1     FRACATEEGYFEVGIWQ--   375
At3g16150   FRGCATEDGFMEVAIWE--   325
PvAspG1     FRACATEDGFMEVAIWD--   326
LjNSE1      FRGCATEDGFMEVGIWE--   326
At5g08100   FRACASEDGYSEIAIWPNN   315
PpASPG2     FRACATEDGFSEVGILA--   305
PpASPG3     FRACATEDGYSEVGILA--   305
            **.**:*:*: *:.*
```

Supplementary Figure S1. Amino acid sequence alignment of plant ASPG proteins. ASPG protein sequences were downloaded from databases www.scbi.uma.es/sustainpinedb/(PpASPG1 = sp_v3.0_unigene4029, PpASPG2 = sp_v3.0_unigene15077, PpASGP3 = sp_v3.0_unigene36994), arabidopsis.org (At3g16150 and At5g08100), phytozome.jgi.doe.gov (PvAspG1 = Phvul.001G025000) and www.kazusa.or.jp/lotus/(LjNSE1 = Lj5g3v0296030.1) and aligned using Clustal W. Gray shaded regions contain amino acids that are implicated in substrate binding (Sub. spec.), activation by K^+ (Activation) or anchored to the L-Asp reaction product docking the substrate (D), as identified by Bejger et al., (2014). Boxed residues constitute a catalytic switch ON/OFF. The arrow indicates the scissile bond that is cleaved during the autoproteolytic activation process. K^+-dependent asparaginases are shaded in red and K^+-independent asparaginases in blue.
