# Supplementary material for: Characterization of Three L-Asparaginases from Maritime Pine (Pinus pinaster Ait.)
Source: Front Plant Sci. 2017 Jun 23;8:1075. doi: 10.3389/fpls.2017.01075 (PMC5481357; doi:10.3389/fpls.2017.01075)
Supplement: Supplementary file 5 [file Image_2.PDF]

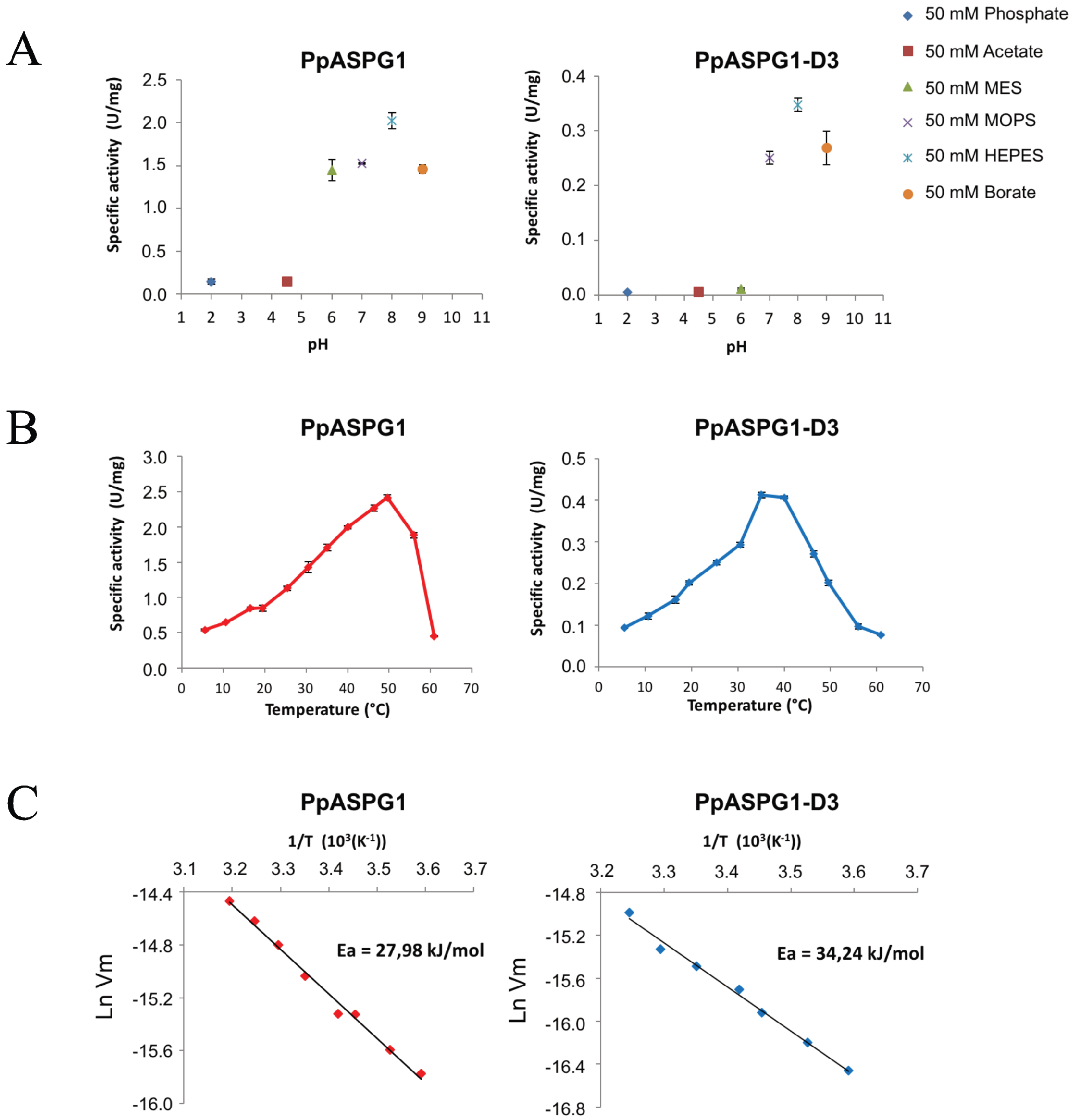

Supplementary Figure S2. Optimum pH (A), optimum temperature (B) and activation energy (C) of PpASPG1 and PpASPG1-D3. Bars represent standard deviation of three replicates.
